# Molecular Insights into Antimicrobial Resistance Traits of Multidrug Resistant Enteric Pathogens isolated from India

**DOI:** 10.1038/s41598-017-14791-1

**Published:** 2017-10-31

**Authors:** Pawan Kumar, Satyabrata Bag, Tarini Shankar Ghosh, Prasanta Dey, Mayanka Dayal, Bipasa Saha, Jyoti Verma, Archana Pant, Shruti Saxena, Anbumani Desigamani, Preety Rana, Dhirendra Kumar, Naresh C. Sharma, Pranita Hanpude, Tushar K. Maiti, Asish K. Mukhopadhyay, Rupak K. Bhadra, G. Balakrish Nair, Thandavarayan Ramamurthy, Bhabatosh Das

**Affiliations:** 1 Molecular Genetics Laboratory, Centre for Human Microbial Ecology, Translational Health Science and Technology Institute, NCR Biotech Science Cluster, Faridabad, 121001 India; 2 School of Life Sciences, Manipal University, Manipal, 576104 Karnataka India; 3 Maharishi Valmiki Infectious Diseases Hospital, Kingsway Camp, Delhi, 110009 India; 4 Regional Centre for Biotechnology, NCR Biotech Science Cluster, Faridabad, 121001 India; 5 National Institute of Cholera and Enteric Diseases, P33 CIT Road, Scheme XM, Beliaghata, Kolkata, 700 010 India; 6 Infectious Diseases and Immunology Division, CSIR-Indian Institute of Chemical Biology, Kolkata, 700 032 India; 7 Present Address: Research Policy and Cooperation Unit, Communicable Diseases Department, World Health Organization (WHO), Mahatma Gandhi Marg, Indraprastha Estate, New Delhi, 110 002 India

## Abstract

Emergence of antimicrobial resistant Gram-negative bacteria has created a serious global health crisis and threatens the effectiveness of most, if not all, antibiotics commonly used to prevent and treat bacterial infections. There is a dearth of detailed studies on the prevalence of antimicrobial resistance (AMR) patterns in India. Here, we have isolated and examined AMR patterns of 654 enteric pathogens and investigated complete genome sequences of isolates from six representative genera, which in aggregate encode resistance against 22 antibiotics representing nine distinct drug classes. This study revealed that ~97% isolates are resistant against ≥2 antibiotics, ~24% isolates are resistant against ≥10 antibiotics and ~3% isolates are resistant against ≥15 antibiotics. Analyses of whole genome sequences of six extensive drug resistant enteric pathogens revealed presence of multiple mobile genetic elements, which are physically linked with resistance traits. These elements are therefore appearing to be responsible for disseminating drug resistance among bacteria through horizontal gene transfer. The present study provides insights into the linkages between the resistance patterns to certain antibiotics and their usage in India. The findings would be useful to understand the genetics of resistance traits and severity of and difficulty in tackling AMR enteric pathogens.

## Introduction

Infectious diseases are one of the major problems in public health and global economies. Bacteria contribute to more than 54% of global infectious diseases^1^. Antimicrobials against bacteria, viruses, fungi and parasites are the most important tools in medicine to prevent and cure microbial infections. They eliminate or inhibit microbial growth by impeding essential cellular processes including DNA, RNA and protein syntheses, cell wall biosynthesis and folate metabolism^2^. Recent reports have shown that the efficacy of numerous, if not all, antimicrobials is severely compromised due to the emergence of antimicrobial resistance (AMR) in pathogens^3–5^.

Resistance to antimicrobials is a natural process and the crisis of resistance is expedited due to the indiscriminate usage of antimicrobial compounds; they eliminate susceptible population and provide a suitable environment and space for resistant variants to flourish^6,7^. The principal mechanisms that confer resistance against antibiotics could be due to two genomic adaptations: spontaneous mutation and/or acquisition of resistance traits by horizontal gene transfer^7,8^. Mutations can alter the target sequence, overexpress target or efflux pumps and reduce intake of antibiotics. Acquired resistance traits can modify the target post-translationally, inactivate antibiotics by hydrolysis or chemical modification, or may provide alternative metabolic pathways or efflux pumps etc^9^.

In India, Infectious diseases are the real threat to public health. The emergence of AMR pathogens such as *Klebsiella pneumoniae, Providencia stuartii, Escherichia coli, Salmonella* Typhi, *Pseudomonas aeruginosa* and several species of *Shigella* resistant to some or all antibiotic classes commonly used in the treatment of bacterial infections have spurred the burden^10–13^. Despite the alarming increase in the prevalence of AMR bacterial pathogens in India, publicly available information regarding the current national picture of resistant pathogens and molecular identity of resistance traits is meager^5,13^. To combat the threat of AMR pathogens, knowledge on molecular identity of resistance traits, their mechanisms of acquisition and dissemination in the microbial community are essential. In the present study, we have analyzed antibiotic susceptibility of 654 Gram-negative enteric pathogens from two Centers in India, one in Kolkata (East India) and other in Delhi (North India). We determined the complete genome sequence of extensively drug-resistant (XDR) enteric pathogens belonging to six different genera. The whole genome sequences provided molecular insights into resistance traits, genetic elements carrying resistance encoding genes and their possible mode of dissemination to the susceptible microbes. Functional characterizations of resistance genes revealed their ability to confer resistance in multiple hosts. The knowledge generated in this study will help to understand the biology of resistance encoding traits in the enteric pathogens of public health importance, and could be helpful in infectious disease management and in the development of strategies to cure resistance-encoding functions from the genome of AMR pathogens.

## Results

### Overall resistance patterns of pathogens isolated from 2009–2015

Six hundred fifty four Gram-negative enteric pathogens belonging to 8 genera (*Escherichia, Shigella, Klebsiella, Salmonella, Providencia*, *Vibrio*, *Pseudomonas, Aeromonas*) and 13 different species isolated between 2009 and 2015 were examined for antibiotic sensitivity (Table S1). The bacterial pathogens were isolated from two Centers in India, one in Kolkata (East India) and the other in Delhi (North India). For the present study, we selected 22 different antibiotics belonging to 9 different classes that interact with essential cellular components of bacteria and interrupt major cellular metabolic pathways including DNA replication and repair, transcription, protein synthesis, folate metabolism and cell-wall biosynthesis.

**Figure 1:**
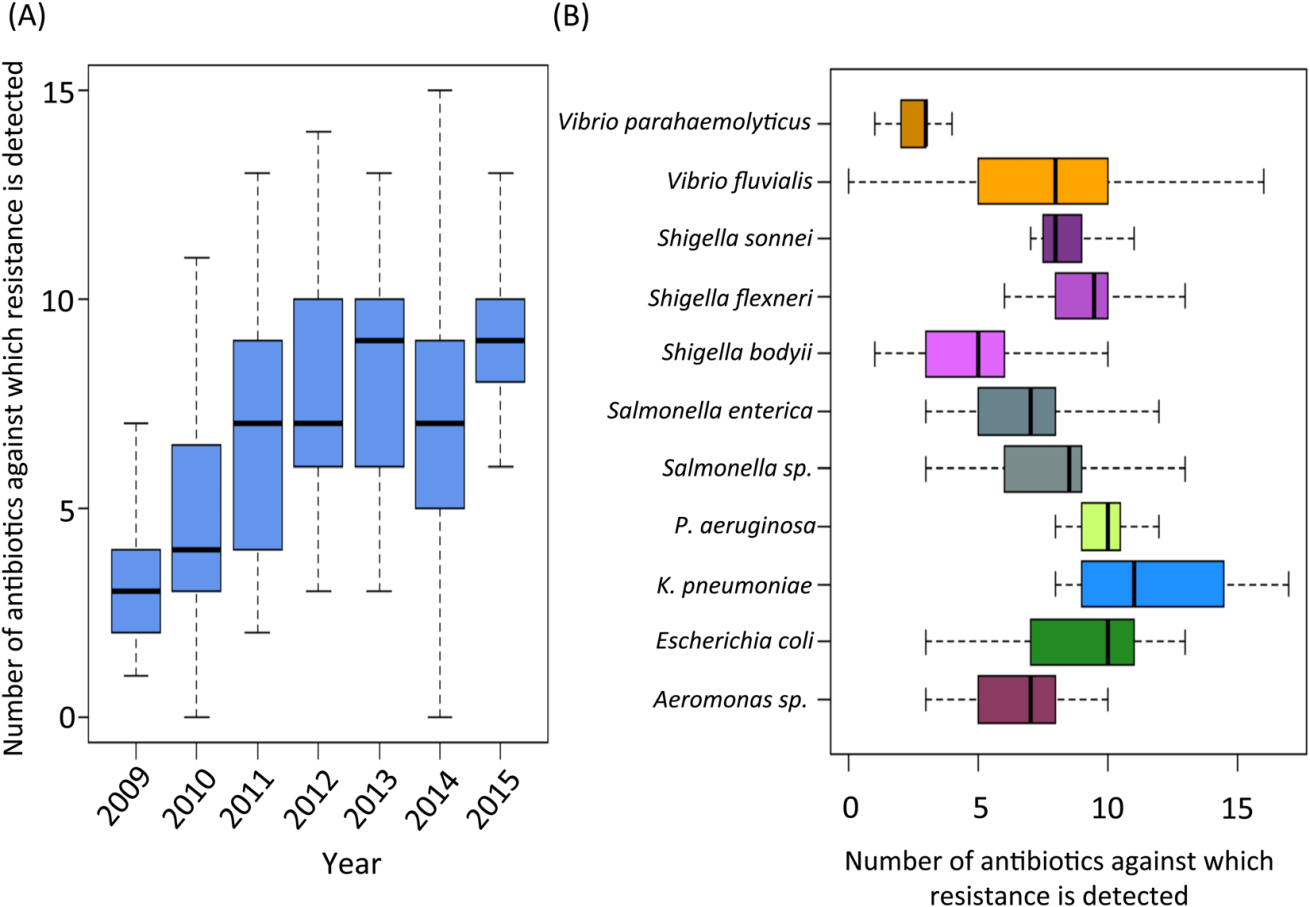
(**A**) Resistance diversity (i.e. number of antibiotics against which resistance is detected) of the isolates detected from 2009–2015. (**B**) Variation of the resistance diversities of the individual pathogens detected in this study. The figure shows the progressive increase of antibiotic resistance in isolates till 2011. After 2011, the resistance diversities of the isolates seem to be more or less invariable. The resistance diversities also show significant variations between isolates belonging to different species.

For each isolate, the resistance/susceptibility profile against all the antibiotics were reported in Table S1. The number of antibiotics, against which the resistance was detected, was referred to as the ‘Resistance Diversity’ for the given isolate. The variation in the ‘resistance diversity’ of pathogens isolated from 2009 to 2015 is shown in Fig. 1A. The resistance diversity of the MDR pathogens was observed to show a progressive increase from 2009 to 2011 (P < 0.014; Mann-Whitney U test). However, 2011 onwards, the resistance diversity was observed to show no significant variation. This is important to note that some of the pathogens like *Pseudomonas aeruginosa*, *Providencia stuartii* showed intrinsic resistance against few selected antibiotics^14^.

Resistance diversity of pathogens was also observed to show species-specific patterns (Fig. 1). While isolates of *K. pneuomoniae* were observed to have the highest resistance diversities, the lowest resistance diversities were observed for the *V. parahaemolyticus* isolates. Within the three species of the *Shigella*, the resistance diversity of *S. bodyii* isolates was observed to be significantly lower as compared to those of *S. flexneri* (P < 0.0093; Mann-Whitney U test) and *S. sonnei* (P < 0.02; Mann-Whitney U test). While *K. pneumoniae* and *V. fluvialis* were observed to show the highest variation in their resistance profiles, isolates of *P. aeruginosa*, *S. flexneri*, *S. sonnei* and *V. parahaemolyticus* were observed to have relatively similar resistance diversities.

### Resistance trends for different antibiotics

**Figure 2:**
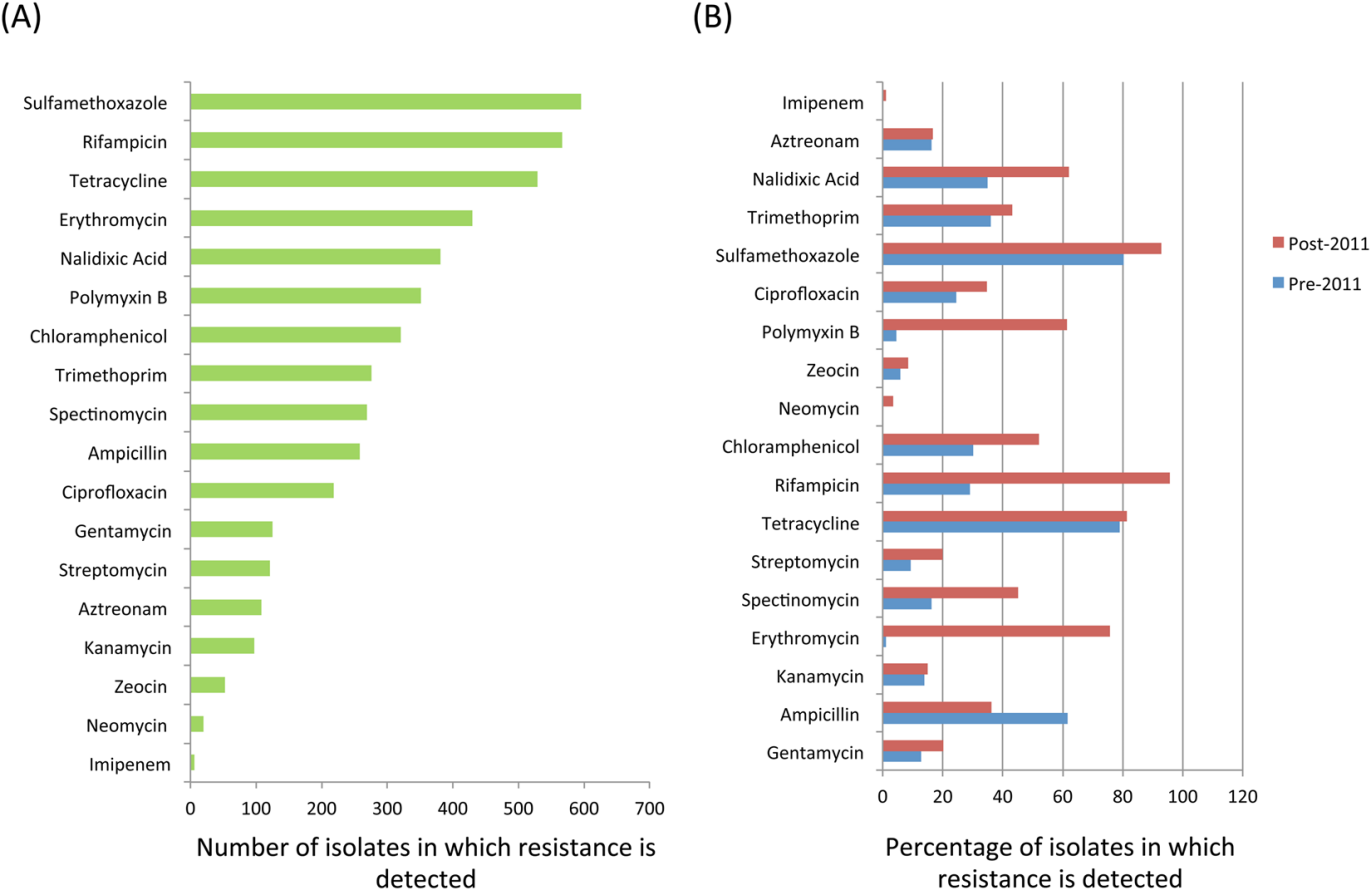
(**A**) Number of isolates in which resistance is detected for each antibiotic. (**B**) Detection percentages of resistance to each antibiotic, detected in pre and post 2011. Besides providing an illustration of the pattern of antibiotic resistances across all MDR isolates, the figure also highlights the rise in resistance to certain antibiotics. In contrast, the resistance to ampicillin (a legacy antibiotic) is observed to have decreased post 2011. Many of these trends correlate with the usage of various antibiotics analyzed in previous studies.

We next profiled the overall trends of resistance against various antibiotics across all isolates over the years (Fig. 2AB). Overall, sulfamethoxazole resistance was observed to be the highest (97%). In addition, resistance to rifampicin, tetracycline, erythromycin, nalidixic acid, polymyxin and chloramphenicol were also observed to be greater than 50% of the isolates (Fig. 2A). On the other hand, resistances to imipenem, neomycin, zeocin, kanamycin and aztreonam were observed to be the lowest among all isolates. Given that the resistance diversities of pathogens were observed to show distinct trends of variation pre- and post-2011, the detection percentage of resistance to the various antibiotics was then profiled separately for the two different time periods (Fig. 2B). Resistances to almost all antibiotics showed an increase post 2011. The only exception to the above trend was ampicillin, the resistance to which was observed to show a significant decrease post 2011 (P < 1.8e-5; Fisher exact test; Supl. Figure S1). On the other hand, for some of the antibiotics, the increase in resistance detection post 2011 was very significant. These included the quinolone antibiotics nalidixic acid (increase from 34% to 62%; P < 2.08e-6;) and ciprofloxacin (24% to 34%; P < 0.06); the aminoglycoside antibiotics streptomycin (9% to 20%; P < 0.016) and spectinomycin (16% to 45%; P < 1.43e-7); the polypeptide antibiotic polymyxin B (4.6% to 61%; P < 1.57e-14) and chloramphenicol (30% to 52%; P < 0.0001). However, the most drastic increase in resistance was observed for the macrolide erythromycin, which increased from 1.1% to 76% from pre-2011 to post-2011 (P < 2.2e-16). The distinct changes in resistance patterns could be reflection of the antibiotic usage trends in India. Previous studies have shown a decline in the usage of ampicillin and increase of usage of quinolone antibiotics^13^. Similarly, in India, Polymyxin usage has also been shown to significantly increase from 2000–2010^15^. These changes in usage patterns are precisely reflected in the resistance trends observed in the current study (Supl. Figure S1). Many of the antibiotics like carbapenems have been used in only post 2010. In line with this trend, in this study, resistance to imipenem (carbapenem antibiotic) is only observed in the isolates post 2011.

### Resistance patterns for the different pathogens

**Figure 3:**
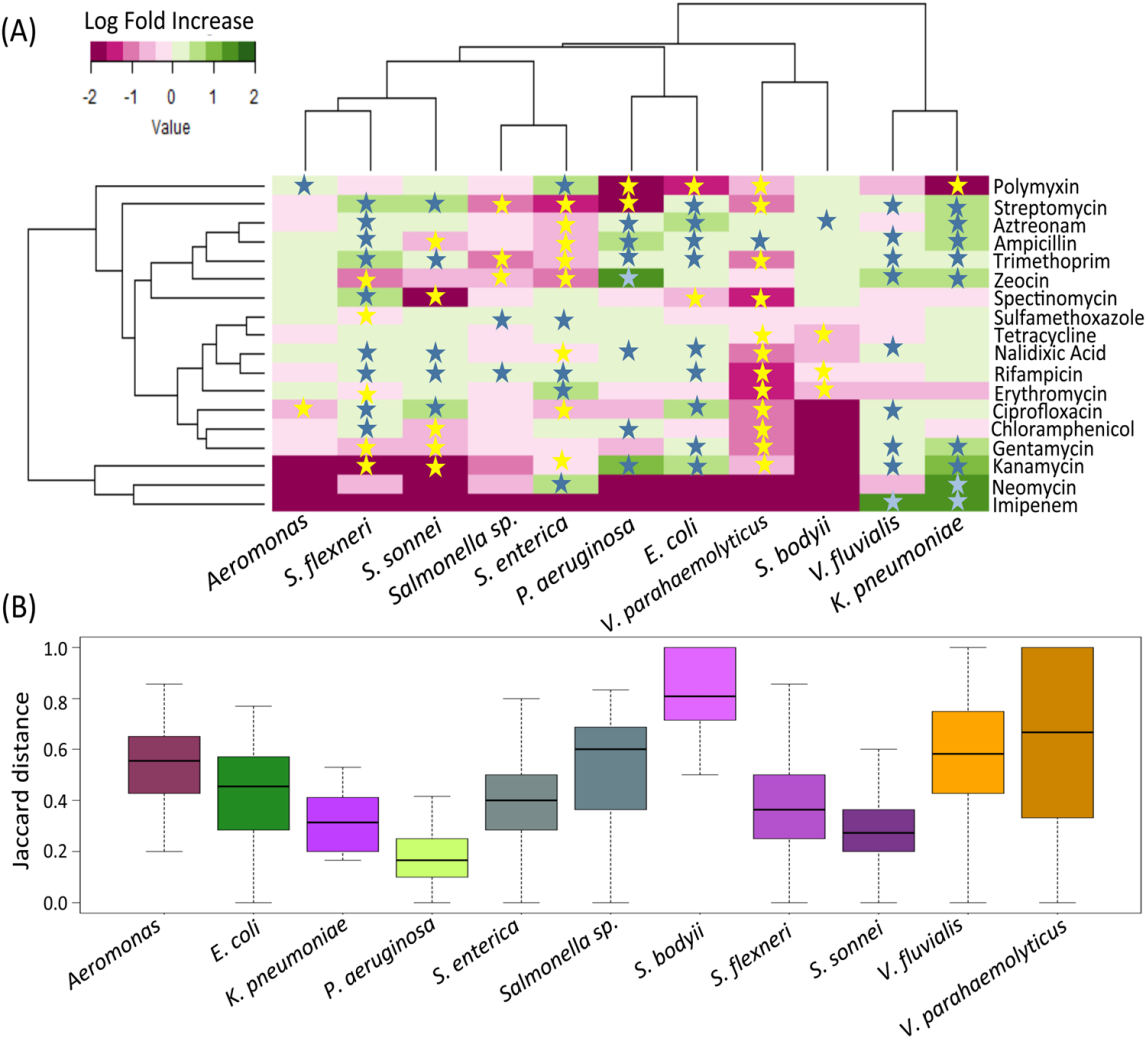
(**A**) Log fold change in the resistance detection to various antibiotics in each of the pathogens (as compared to all others). Only species with more than one isolates (detected in the current study) were included in this analysis. While antibiotics against which resistances are significantly high and significantly low (in the different pathogens) are indicated in blue and yellow stars, respectively. (**B**) Variation of pairwise Jaccard distances between the resistance profiles of the isolates belonging to the various pathogens. Different species are not only observed to have distinct resistance patterns, but the isolates within species are also observed to vary to different extents in their resistance profiles.

The resistance traits specific for the different pathogens were then investigated to identify associations between resistances to various antibiotics in different pathogenic species. Amongst the 11 different MDR pathogens, *V. parahaemolyticus* was observed to have the lowest detection rates of resistances to various antibiotics. However, ampicillin resistance was observed to be significantly high (P < 7.2e-5) in *V. parahaemolyticus* (Fig. 3A). The resistance to several antibiotics including gentamycin, kanamycin, erythromycin, spectinomycin, tetracycline, chloramphenicol, ciprofloxacin, sulfamethoxazole, trimethoprim and nalidixic acid were observed to be significantly low as compared to the other MDR pathogens (P < 0.05) (Fig. 3A). *Aeromonas* was observed to have a significantly high resistance to polymyxin (P < 0.047) (Fig. 3A). Two species of Shigella namely, *S. flexneri* and *S. sonnei* were observed to have similar pattern of resistances. Both were observed to have significantly high detection of resistances (P < 0.05) to antibiotics ciprofloxacin, nalidixic acid, trimethoprim and streptomycin, and low detection rates for the aminoglycoside based antibiotics gentamycin and kanamycin (Fig. 3A). However, while *S. flexneri* was observed to have a significantly high detection rate for ampicillin resistance and low detection rate for resistance to chloramphenicol, *S. sonnei* was observed to show the exact opposite trend.

*E. coli* isolates had significantly high detection rates for resistances to antibiotics gentamycin (P < 0.005), ampicillin (P < 4.7e-7), kanamycin (P < 5.3e-9), streptomycin (P < 0.01), trimethoprim (P < 0.005), nalidixic acid (P < 1.3e-8), aztreonam (P < 1.1e-5) and ciprofloxacin (P < 2e-16). Around 90% isolates of *E. coli* were resistant to ciprofloxacin (as compared to a detection rate of 30% across all 654 isolates). On the other hand, resistances to spectinomycin and polymyxin B were observed to have significantly low detection rates in *E. coli* isolates (P < 7.9e-5, P < 1.3e-10, respectively). Similarly, isolates of *P. aeruginosa* had significantly high resistance to the antibiotics ampicillin (P < 7.1e-7), kanamycin (P < 1.5e-13), chloramphenicol (P < 2.04e-5), zeocin (P < 2.2e-16), trimethoprim (P < 4.51e-5) and nalidixic acid (P < 3e-4). As observed for *E. coli*, polymyxin B resistance was observed to be significantly low in the isolates of *P. aeruginosa* (P < 7.608e-6). *K. pneumoniae* and *V. fluvialis* were also observed to have similar trends in their resistance patterns (Fig. 3A). For both these pathogens, the MDR isolates were observed to have significantly high resistances (P < 0.05) for the antibiotics streptomycin, ampicillin, trimethoprim, zeocin, gentamycin, kanamycin and imipenem. Interestingly, imipenem resistance was observed only in the isolates of *K. pneuomoniae* and *V. fluvialis*. Out of the six isolates of imipenem resistance, five were observed to belong to *V. fluvialis*. The only differences were that, while *K. pneumoniae* isolates were observed to have significantly high detection rates of resistance to the antibiotics aztreonam (P < 0.016) and neomycin (P < 0.005), detection rates of nalidixic acid resistance and ciprofloxacin resistance were observed to be significantly high for isolates of *V. fluvialis* (P < 0.001 and P < 0.001, respectively). *K. pneumoniae* isolates were also observed to have significantly low detection rates for resistance against polymyxin (P < 0.04).

*S. enterica* isolates, on the other hand, were observed to have a resistance pattern distinct from the other pathogenic species detected in the current study. A key differentiating feature of these isolates (in contrast to other pathogens like *E. coli*, *P. aeruginosa* and *K. pneumoniae*) was the significantly high rates of polymyxin B resistance (84% as compared to 27% in other pathogens; P < 2.2e-16). Additionally, resistance detection rates against streptomycin, aztreonam, ampicillin, trimethoprim, ciprofloxacin and kanamycin were observed to be significantly low (all P < 1e-10). Besides this, significantly high resistance rates were also observed for the antibiotics sulfamethoxazole, rifampicin, erythromycin and neomycin.

### Intra-species variation in resistance profiles

In addition to identifying specific patterns of high or low resistance against certain antibiotics, we also checked whether isolates from different species might also display different degrees of intra-species variation in their resistance profiles. For this purpose, isolates of different species were grouped separately and pairwise Jaccard distances between the resistance profiles (of the isolates) in each group (Fig. 3B).

The lowest Jaccard distances were observed between the isolates of *P. aeruginosa* indicating a high degree of similarity between the isolates of this species (Mann-Whitney U test P-value < 0.001). In contrast, isolates of *S. bodyii* were observed to have the highest variation in their resistance profiles. Interestingly, the isolates of both *V. parahaemolyticus* and *V. fluvialis* were not only observed to have relatively higher distances between their resistance profiles (Mann-Whitney U test P-value < 1e-8 and < 2.2e-16, respectively), but also the variation in Jaccard distances was observed to be much greater, indicating isolates within these species formed distinct clusters, with each cluster having distinct resistance profiles.

### Co-detection patterns of antibiotic resistances

**Figure 4:**
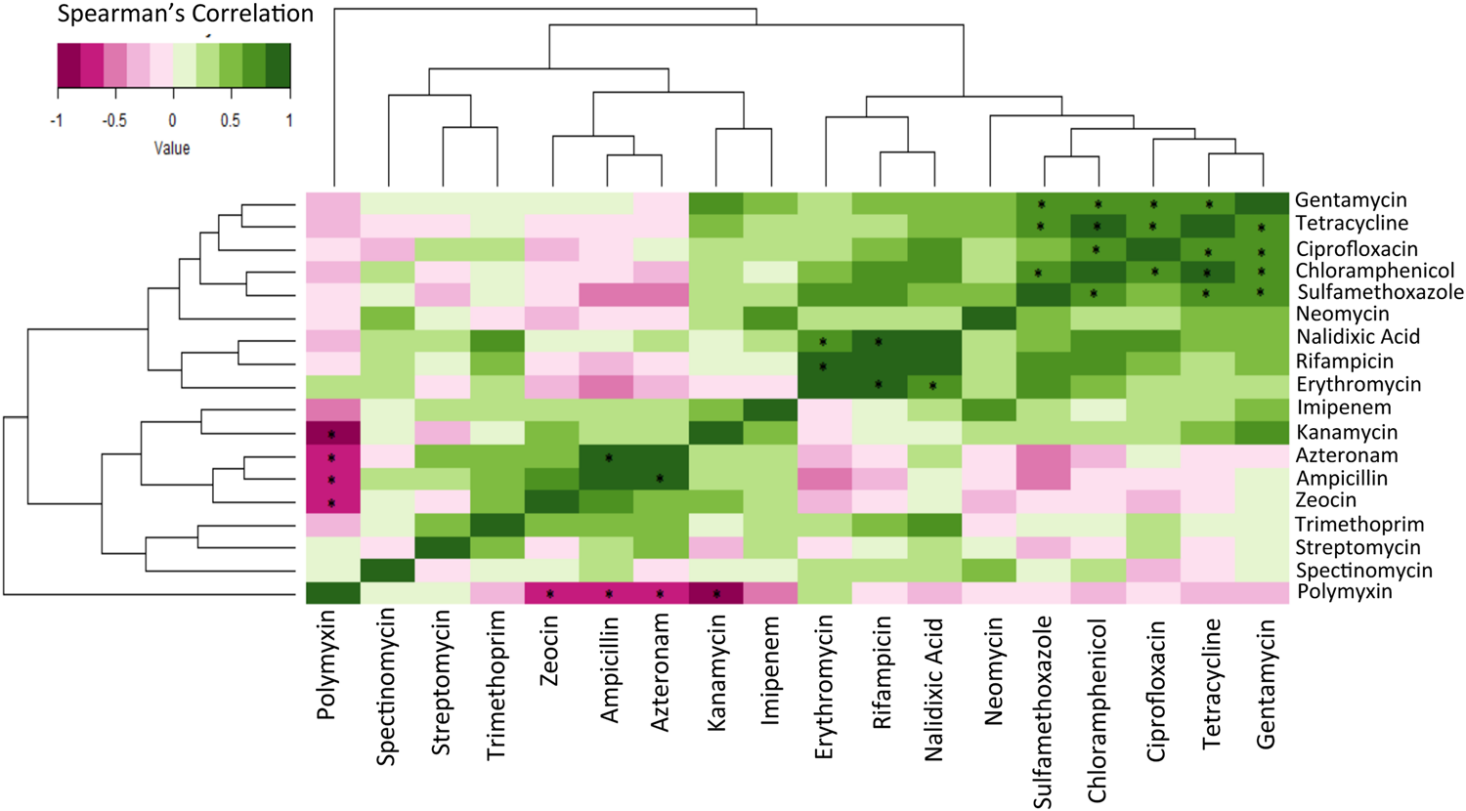
Correlation in the resistance trends of various antibiotics. The figure indicates groups of antibiotic that have similar patterns of resistances across the 11 different pathogens, for which more than 1 isolates were detected in the current study. Two species, *P. stuartii* and *S. dysenteriae*, which contained just one isolate were not included in the statistical/association analyses. Correlations with P-value of less than 0.10 corrected for multiple comparisons using Benjamini-Hochberg procedure are highlighted using*.

We also examined the correlation between the detection rates of resistance to various antibiotics across the pathogens (Fig. 4). For antibiotics having a strong positive correlation in their resistance detection rates, genetic traits conferring resistance to these are highly likely to be present or transferred together across different sets of bacteria. Based on the positive correlation patterns, the antibiotics could be categorized into four major groups (Fig. 4). A majority of these co-occurrence or co-inhibition trends were observed to be statistically significant (P-value < 0.1 corrected using Benjamini-Hochberg procedure). The first group contained the antibiotics gentamycin, tetracycline, ciprofloxacin, chloramphenicol, sulfamethoxazole and neomycin. This was the most dominant group amongst all antibiotics. Resistance detection rates of these antibiotics were observed to not only have strong positive correlation amongst each other, but also marginally high correlations with several other antibiotics like nalidixic acid, rifampicin, erythromycin and kanamycin. The second group was the triad of nalidixic acid, erythromycin and rifampicin (the resistance rates corresponding to these antibiotics having extremely strong positive correlation amongst each other). The third group consisted of aztreonam, ampicillin and zeocin. Besides having a highly positive correlation in the resistance detection rates (amongst each other), this group was observed to have none or even negative correlations with the resistance detection rates of the first two groups of antibiotics (especially sulfamethoxazole, neomycin, chloramphenicol, erythromycin). This indicates that the genomic elements conferring resistances to these antibiotics have distinct presence and mechanisms of transfer as compared to the first two groups. The fourth group contained the pair streptomycin and trimethoprim. Polymyxin resistance on the other hand showed detection pattern that was the least correlated with the resistance patterns of all other antibiotics.

### Genomics of XDR enteric pathogens

We analyzed the genome of XDR pathogens to explore the molecular identities of resistance traits and the genetic elements that carry the resistance genes. Out of 654 isolates, 27 pathogens (4.1%) belonging to seven genera displayed XDR phenotypes (sensitive against only one or two classes of antibiotics), with resistance against ≥14 antibiotics (Table S2). We selected one representative isolate from each genus (Table S2) showing highest number of resistance, except *S. flexneri*, against 9 different classes of antibiotics. The complete genome sequences of these six XDR isolates were obtained by next generation DNA sequencing and investigated for multiple resistance traits and MGEs that are physically linked with the resistance traits. Deep sequencing of the complete genome of six XDR pathogens was accomplished by using GS 454 GS FLX+pyrosequencer that yield more than 50X coverage for each genome (Table 1). Average outputs, read lengths and number of reads in each of the genomes are summarized in Table 1. Encoded functions and relevant characteristics of each of the six XDR genomes are summarized in Table S3. We observed similar genome size, G+C content, codon usage and genome characteristics of all the sequenced genomes to their previously sequenced model counterparts of the same species^16–19^. Each annotated resistance gene was examined individually for identity with well-characterized AMR gene sequences deposited in GenBank, ARDB and PDP databases (Supl. Table S5A).

**Table 1:** Relevant whole genome sequencing information of six XDR isolates characterized in this study.

Characteristics	*E. coli (EPEC)*	*S. flexneri*	*P. aeruginosa*	*S*. Typhimurium	*K. pneumoniae*	*P. stuartii*
Strain ID	MV292587	IDH07210	MV36846	MV32691	MV36808	MV493
Sequence size (Mb)	430	207	303	363	390	429
No. of reads	592054	428839	490091	563297	553858	586289
Avg. read lengths (bp)	727	483	620	646	704	732
No. of scaffolds	61	307	65	29	93	76
GC content (%)	50.5	50.5	66.3	52.2	56.6	41.0
Mean seq. size	48264.3	5769.2	56033.5	68477.5	33376.8	62370.5
Longest scaffold size (bp)	541956	75239	591202	667925	365674	278521
Total number of ORF	5429	4664	6117	4817	5767	4597
ORFs encode Virulence, toxin and AMR	128	116	145	121	136	29
Phage, Tn, plasmids	150	42	8	25	113	41

The genome of all the sequenced XDR pathogens comprises one circular chromosome and multiple extrachromosomal genetic elements (Table 1). The number of ORFs predicted in different genomes varies from 4597 to 6117 (Table 1). In all the sequenced genomes the number of genes involved in virulence, toxin production, disease development and antibiotic resistance are varied widely depending on the type of isolates (Table 1). The highlights of these findings are summarized in the subseqent sections.

**Figure 5:**
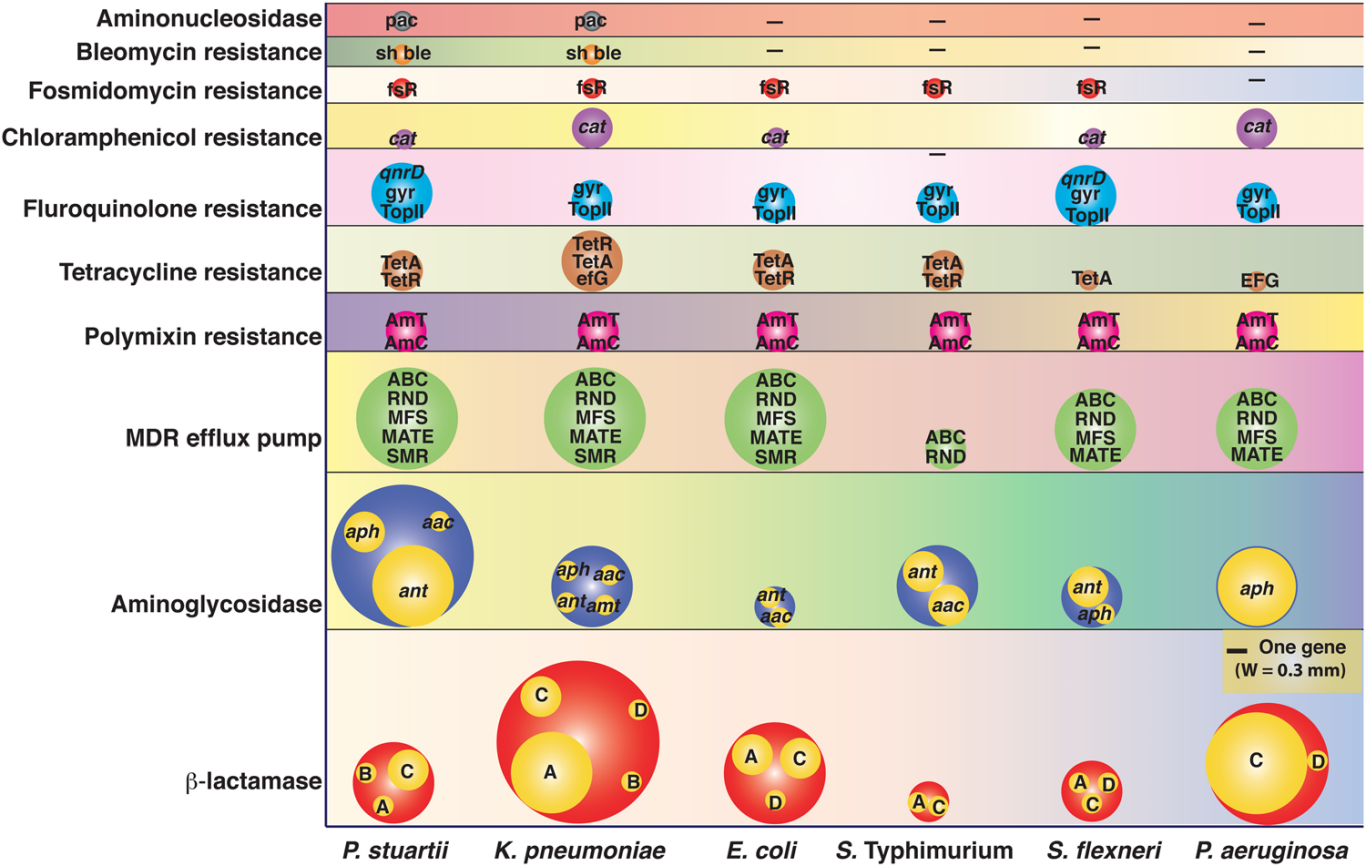
Antimicrobials resistance encoding genes in the XDR isolates. Bubble sizes correspond to the number of genes detected in the genome of isolates. Subclasses of the resistance function for each category are also mentioned inside the bubble. The picture was drawn to the scale. A, C and D denote serine-β-lactamases, whereas B denotes metallo-β-lactamase. *aph* = *Aminoglycoside phosphotransferase, aac* = *aminoglycoside acetyltransferase, ant* = *aminoglycoside nucleotidyltransferase, amt* = *aminoglycoside resistance methyltransferase, ABC* = *ATP-binding cassette transporter, RND* = *Resistance nodulation division, MFS* = *Major facilitator superfamily, MATE* = *Multidrug and toxic efflux, SMR = Small multidrug resistance, pmrJLM* = *Proteus mirabilis polymyxin B resistance encoding operon, pmrBD* = *Polymixin B resistance genes, TetA* = *Tetracycline resistance protein A, TetR* = *Tetracycline resistance regulatory protein R, EFG* = *Elongation factor G, qnrD* = *Quinolone resistance, gyr* = *Gyrase, topII* = *Type II topoisomerase, cat* = *chloramphenicol acetyltransferase, fsr = Fosmidomycin resistance protein, sh ble* = *Bleomycin resistance gene, pac* = *Puromycin N-acetyl-transferase*.

The resistance against antibiotics can be intrinsic or acquired via mutations in chromosomal genes and by horizontal gene transfer. Whole genome sequence analysis of the XDR pathogens revealed more than 23 genes linked to the resistance functions, which is in agreement with the antimicrobial susceptible results for each of the pathogen (Fig. 5). Resistance genes from different classes, including intrinsic drug resistance, efflux systems, antibiotic modifying or degrading enzymes, target-altering enzymes were identified in almost all the sequenced genomes (Fig. 5).

### Diversity and abundance of antibiotic resistance traits in the genome of XDR isolates

Analyses of the genomes of six XDR isolates revealed important differences in the diversity and abundance of resistance traits. The genome of the each XDR isolate carried multiple resistance genes against β-lactam and aminoglycoside antibiotics, besides possessing multiple multidrug resistance efflux pumps (Fig. 5). The highest numbers of resistance genes were detected against β-lactam antibiotics (Fig. 5; Supl. Table S5A). A single isolate of *K. pneumoniae* (MV36808) carried eight different *bla* encoding genes, representing all four Ambler classes (A, B, C, and D) of *bla* genes. Similarly, *P. aeruginosa* isolate 36846 harbored six different *bla* genes. Two and three different *bla* alleles were detected in the genome of *S*. Typhimurium MV32691 and *S. flexneri* MV07210, respectively. Highest numbers of aminoglycoside resistance genes were detected in the genome of *P. stuartii* MV493 followed by *K. pneumoniae* MV36808, *S*. Typhimurium MV32691, *P. aeruginosa* 36846, *Sh. flexneri* MV07210 and *E. coli* MV292587. It is important to note that, a single isolate can harbor multiple resistance genes against each class of antibiotic.

### Characterizations of resistance genes and genomic elements linked with the resistance traits

Whole genome sequence analysis of six XDR isolates revealed presence of multiple genes that are presumably associated with antibiotic resistance. However, in the above analysis, the functions of AMR genes are simply inferred by comparing sequence homology to the genes that have been catalogued as resistance genes in the databases. Therefore, to rule out the possible false-positive annotation, we validated functions of all the genes encoding enzymes that contribute to the resistance phenotype of an isolate by either degrading or modifying antibiotic scaffolds. We amplified 12 different genes from the genome of *P. stuartii* and *K. pneumoniae* and cloned them into replicative or integrative expression vector pBD62 and/or pBAD24 and determined their function in homologous and heterologous hosts (Supl. Table S5B). Most of the genes selected for functional evaluation confirmed resistance against reported MIC of the respective antibiotics in both homologous and heterologous hosts (Supl. Table S5C). We also observed that *bla*_*NDM*_, which encodes metallo-betalactamase, is active against all the tested β-lactam antibiotics, except aztreonam. The activity of the *bla*_*NDM*_ encoded enzyme was completely inhibited in the presence of metal ion chelating substance EDTA. In this context, it is important to mention that multidrug resistance efflux pumps are ubiquitous in the XDR isolates, but we did not validate functions of the predicted efflux pumps because of the technical complications to reconstruct the multi-component functional efflux unit in heterologous hosts.

**Figure 6:**
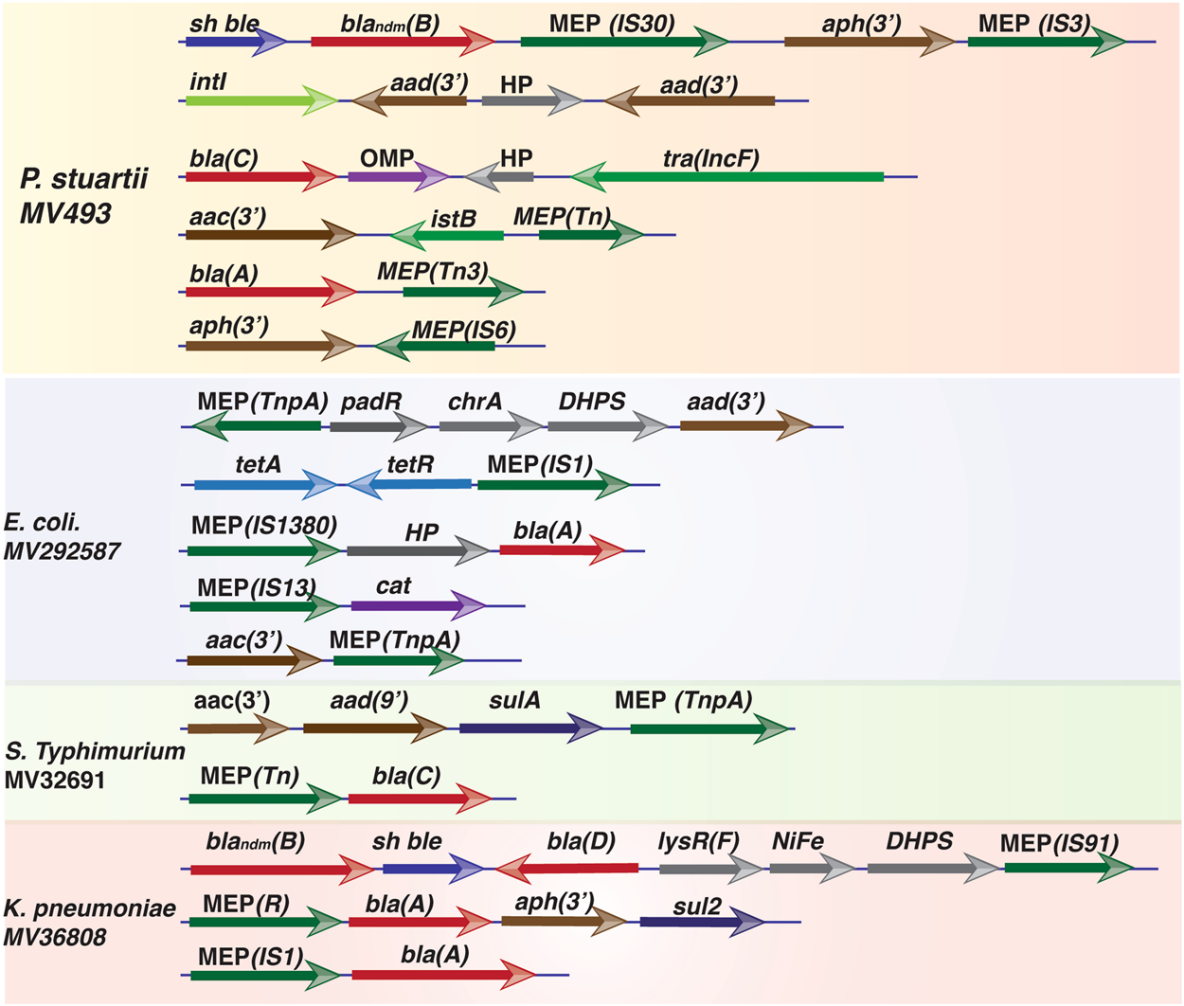
Schematic representation of different antimicrobial resistance encoding genes and their physical association with mobile genetic elements. Genomes of four XDR isolates were scanned to identify the link between resistance genes and mobile element proteins. Description of the resistance genes is provided in the legend of Fig. 5. HP = Hypothetical protein, *chrA* = Chromate transport protein, *padR* = Phenolic acid decarboxylase-regulator, MEP = Mobile element protein, OMP = Outer membrane protein, *tra* = transfer protein, *sulA* = Sulfonamide resistance gene, DHPS = Dihydropteroate synthase, *IstB* = Transposon NTP-binding protein, *intI* = Integrase, TnpA = Transposase, IS = Insertion sequence.

The genome of all the sequenced XDR isolates harbors several MGEs such as plasmids, transposons, bacteriophages, integrative conjugative elements, integrons and genomic islands (Table 1, Table S3). Several MGEs are physically linked with resistance traits (Fig. 6). For instances, two genes, *bla*_*NDM*_ and *sh ble*, encoding resistance against imipenem and zeocin, respectively, in XDR isolates *P. stuartii* MV493 and *K. pneumoniae* MV36808 are physically linked with IS*Aba*125, a IS30 family MGE. Extensive analysis of the genomes of four XDR isolates revealed that several *bla* genes, belonging to Ambler class A-D, often linked with mobile element proteins. Similarly, genes encoding resistance against several members of aminoglycoside are also frequently linked with mobile element protein encoding genes and gene cassette capturing integron integrase (Fig. 6).

The genetic elements that are physically linked with the resistance genes capable of transferring resistance traits to a susceptible host through horizontal gene transfer. To confirm the mobility of the resistance genes, we used naturally competent *V. cholerae* cells SB8 (Supl. Info. S1; Supl. Figure 2) and incubated the strain with the genomic DNA of XDR *P. stuartii*. We observed efficient dissemination of *sh ble* and *bla*_*NDM*_ alleles (1.3 × 10^2^ CFU/μg DNA) to the susceptible host SB8 after overnight incubation in chemically defined growth medium (Supl. Info. S1).

## Discussion

Infectious diarrhea is one of the major public health burdens in low and middle-income countries^20^. Bacterial pathogens, more precisely Gram-negative Proteobacteria, account for the most severe forms of infectious diarrhea including cholera, shigellosis, dysentery and other forms of gastroenteritis. The usual therapeutic approaches to treat diarrheal patients are rehydration and antibiotic treatment^21^. Most of the clinically used antibiotics interfere with cell-wall biosynthesis, DNA replication, transcription, protein synthesis or folate metabolism^9^. Although many of these antibiotics have been used to treat gastroenteritis, in several cases, antimicrobial therapy increases the incidence and duration of intestinal carriage of the pathogens. Recent findings on AMR resistance revealed that resistances to frontline antimicrobials among enteric pathogens are very common and the resistance can spread from multiple sources through a number of pathogenic or commensal microbes by horizontal gene transfer^5,22^. Analysis of the genome sequences of clinical isolates and microbial consortia (metagenomics) led to interesting findings on resistance traits and genetic elements carrying resistance-encoding functions. In enteric bacteria most of the resistance traits are physically linked with autonomously replicating or integrative MGEs and could efficiently transfer to the bacteria that are not in parent-offspring relation^7^.

Development of AMR is an outcome of complex microbial interactions and resistance may arise by acquisition of de-novo mutation during clinical antibiotic treatment or commonly by acquisition of integrative or replicative MGEs that have evolved over time in microbes in the natural ecosystem. The environmental reservoirs of resistance genes are widely diverse and similar resistance genes may present in distantly related bacterial species, which can co-exist in the same habitat^23,24^. It was reported that *K. pneumoniae*, *V. cholerae*, *E. coli*, *P. aeruginosa, S*. Typhi and *P. stuartii* are naturally competent and can uptake naked DNA from the environment in suitable conditions^25–27^. In this study, we also observed that *V. cholerae* can efficiently uptake naked genetic elements encoding resistance function in the presence of chitin, a nitrogenous polysaccharides which is the major component in the exoskeleton of crustaceans. Although conjugation and transduction are also efficient in the spread of MGEs, both have some limitations in cross-species DNA exchange. Since, uptake of free DNA has no such limitation, we believe that transformation plays important role in the acquisition and dissemination of resistance genes between enteric pathogens.

The present comprehensive study, to the best of our knowledge, is the first attempt to profile resistance traits among several clinical isolates from India over a period of several years and investigates the molecular identity of resistance traits and explore the possible mechanisms of dissemination among bacterial cells. Antimicrobial susceptibility assays of 654 clinical isolates revealed presence of resistance traits against both synthetic and natural antimicrobial compounds. Several acquired antibiotic resistance traits, which are highly mobile in nature, were detected in the AMR pathogens that can efficiently inactivate or pump out more than nine different classes of antibiotics including β-lactams, aminoglycosides, amphenicols, quinolones, glycopeptides, sulfonamides, tetracyclines, macrolides, and pyrazinamide. These antibiotics have a wide spectrum of targets, which can inhibit multiple essential cellular pathways including protein synthesis, DNA replication, transcription, cell wall biosynthesis and folate metabolism^24^. We observed trends wherein, the prevalence of antibiotic resistance traits in the current isolates of enteric pathogens correlate well with routinely prescribed antimicrobials.

The chronological analysis of resistance patterns revealed that the resistance diversity of enteric pathogens was significantly increased in post 2011 (Fig. 1). Overall, the resistance against sulfamethoxazole is very high (596 out of 654 isolates are resistant), whereas the lowest resistance was observed against imipenem. Similarly, more than 50% of the isolates are resistant to rifampicin, tetracycline, erythromycin, nalidixic acid, polymyxin and chloramphenicol, which are most frequently prescribed antimicrobials in the developing countries. Increased resistance to several antibiotics is observed to have direct correlations with the consumption trends of several antibiotics in India. Most commonly prescribed drugs in diarrheal patients are sulfamethoxazole-trimethoprim, β-lactams (cephalosporins) and fluoroquinolones and the proportion of resistance against these drugs has increased considerably^28^.

Whole genome sequence analysis of XDR isolates revealed that resistance is common across the pathogens and almost all the resistance traits are linked with acquired MGEs. More detailed analysis of the genomes showed the presence of multiple resistance traits against each class of antimicrobial scaffolds (Fig. 5). A meta-analysis of the genomes of all the sequenced XDR isolates identified resistance alleles from 29 different classes that can inactivate or pump out antibiotics from their cytosol. Highest numbers of resistance alleles were detected for the β-lactamase enzyme (Fig. 5). The acquisition of metallo-β-lactamases is a primary contributor to the emergence of carbapenem resistant Gram-negative pathogens that threatens the use of several antibiotics including penicillin, cephalosporin and carbapenem. Most of the sequenced XDR pathogens harbor 3-4 different classes of β-lactamases including metallo-β-lactamases (Class B). Highest numbers of β-lactamase encoding genes were observed in the genome of *K. pneumoniae* (Fig. 5). The genome of a single isolate of *K. pneumoniae* (MV36808) harbored 8 different β-lactamase encoding genes. By analyzing the immediate genetic vicinity of the *bla*_*NDM*_ gene of *K. pneumoniae* (MV36808) and *P. stuartii* (MV493) we observed that bleomycin resistance encoding *sh ble* gene is physically linked with the *bla*_*NDM*_ gene (Fig. 6). Further analysis of the genomic scaffolds of these two isolates revealed that the *sh ble* and *bla*_*NDM*_ genes are co-expressed under the control of the same promoter, located upstream of the *bla*_*NDM*_ gene and at the extremity of the insertion sequence IS*Aba125* (Fig. 6). Phenotypically, these isolates showed resistance against both imipenem and zeocin. The resistance traits are functional in homologous and heterologous genetic backgrounds and could disseminate to susceptible bacterial cells by natural transformation.

Like β-lactamases, multiple traits conferring resistance against aminoglycoside antibiotics are ubiquitously detected in all the sequenced genome of XDR pathogens^25^. This correlates well with the overall increase in aminoglycoside usage in clinical facilities across India over the years^15^. Although maximum number of β-lactam resistance genes were detected in the genome of XDR *K. pneumoniae, P. stuartii* genome encodes highest numbers of aminoglycoside antibiotic resistance genes from all three major classes: aminoglycoside phosphotransferase (*aph*), which transfer the phosphoryl group from ATP; aminoglycoside acetyltransferase (*aac*), which transfer the acetyl group from acetyl-CoA; and aminoglycoside nucleotidyltransferase (*ant*), which transfer a nucleotide triphosphate.

The development of resistance against antimicrobials is a natural evolutionary phenomenon and cannot be prevented^7^. The process of emergence of resistant microbes is highly complex and gets influenced by multiple environmental factors including the massive use of antibiotics^25^. The antibiotic resistance crisis and emergence of XDR pathogens can be slowed down if we generate sufficient knowledge on the real time evolution and ecology of resistance pathogens by initiating intensive research on AMR of priority pathogens^27^. To tackle the resistance crisis, the immediate need is rapid identification of drug resistance at point-of-care. Currently, other than culture based sensitivity assays, no universally accepted method has been developed for rapid diagnosis of AMR traits in clinical settings. The findings of the present study provide important information about the prevalence of resistant pathogens and molecular identity of resistance traits. It will be useful to develop molecular diagnostic tools by assimilating identity of resistance traits in the current XDR isolates of India. Information on specific associations of resistance trends against each antibiotic may not only aid in the formulation of pathogen-specific or region-specific treatment regimens, but also designing robust surveillance programs restricting improper antibiotic usage. It is important to note that the isolates from certain pathogens were only detected in specific years (Supl. Figure S3A). We also observe that over the years, the number of types of resistant pathogens from which the isolates were obtained increased from 2009–11 to 2013–15. For example, isolates of pathogenic Shigella and Aeromonas were detected only after 2014. *E. coli, K. pneuomoniae* and *P. aeruginona* were observed only in 2013 (and not before that). There were however two species, namely *V. fluvialis* (detected from 2009–2015) and *S. enterica* (detected from 2012–2015), that were detected across multiple years. In the case of *V. fluvialis*, the number of antibiotics against which resistances were detected showed a significant increase in 2014-15 as compared to 2008-09 and 2010-11 (Supl. Figure S3B) (dunns test P-value < 0.01 for both). Similarly, for *S. enterica* (with the exception of 2014) the number of antibiotic against which resistance was detected was significantly higher in 2015 as compared to 2012 and 2013 (Supl. Figure S3C) (dunns test P-value < 0.001 and <0.01, respectively). These observations indicate that over the years, the MDR isolates not only became diverse in terms of their species types, but also the resistance of certain species has shown an overall increase albeit with fluctuations.

## Conclusions

In the last 10 years, more than 67000 studies have been reported from across the globe on antimicrobial resistance to understand the prevalence of AMR pathogens, diversity and abundance of resistance traits, mechanisms of acquisition and dissemination of resistance genes, as well as on drug-susceptibility testing, rapid diagnosis of AMR pathogens and developing strategy to re-sensitize the resistance variants against existing drugs of public health importance. Despite the availability of the plethora of information on the fundamental science of resistance biology, the information on the rise of resistant pathogens across the globe is surprisingly scarce. Our data show that the clinical isolates of enteric pathogens are continuously evolving to counterbalance the bactericidal/bacteriostatic effects of clinically important antimicrobial drugs because of the remarkable plasticity of their genome. Most importantly, the resistance machineries are also shifting from canonical stable antibiotic target modifications to acquisition of mobile resistance traits that efficiently make the drugs ineffective and could disseminate rapidly to the other microbes in hospital and community settings. To combat the serious threat of rising AMR in enteric pathogens and to stem the decline in effectiveness of antibiotics of public health importance, it is imperative to develop strategies for robust surveillance, restriction on improper antibiotic usage and identify the routes that are facilitating the rapid dissemination of antibiotic resistance in pathogenic and nonpathogenic bacterial cells.

## Materials and Methods

### Bacterial strains and plasmids

AMR profile of all the isolates is given in Table S1. All these isolates, except *P. stuartii*, are from patients with acute diarrhea. Relevant characteristics recombinant plasmids used in this study are listed in Supl. Table S5B. Bacterial isolates were grown under shaking or static conditions at 37 °C in Luria-Bertani (LB) or Mueller-Hinton (MH) Agar medium (Sigma, USA). Different antibiotic discs (BD, USA) or solutions used in this study are mentioned in Supl. Table S5D. Expressions of different genes encoding antibiotic resistance were under the regulation from an arabinose-inducible promoter.

### Isolation, cultivation and identification of enteric pathogens

The following criteria were used for the inclusion of isolates in the current study. First, the isolates were of naturally competent Gram-negative enteric pathogens isolated frequently from the fecal samples of diarrheal patients from different parts of India. The selected isolates are notorious for drug resistance and dissemination of resistance traits to closely and distantly related bacterial species through horizontal gene transfer. Second, the pathogens may link with community or hospital associated infections. Third, the pathogens could be cultivated in BSL2 laboratory and have minimal doubling time. We have not included any Gram-positive enteric pathogens or bacteria that may infect through aerosol or require BSL3 level safety for isolation and drug susceptibility characterizations. Besides the above criteria, there was no bias in the inclusion of any specific species across any year.

Different species of the family Vibrionaceae including *V. parahaemolyticus* and *V. fluvialis* were isolated on thiosulfate-citrate-bile salts-sucrose (TCBS) Agar (Eiken, Japan). Bacterial isolates belonging to family Enterobacteriaceae including *E. coli*, *S. flexneri*, *S. boydii*, *S. sonnei*, *K. pneumonia*, were isolated on MacConkey Agar (Fluka, USA). Sets of biochemical and serological tests were performed to identify isolates. The precise phylogenetic identity of all the whole genome sequenced XDR isolates were first confirmed by 16S rRNA gene sequencing. Bacterial species used in this study were isolated and characterized in biological safety cabinet Class II Type A2 (Logic, LABCONCO, USA).

### Antibiotic susceptibility tests

Thirteen different bacterial species belonging to eight genera were used in the antibiotic susceptibility assay (Supl. Info. S1). Antimicrobial drug susceptibility testing was performed using the disc diffusion as well as broth dilution methods with commercially available antimicrobial discs (Becton Dickinson, Sparks Glencoe, MD, USA) and antibiotics solution respectively, according to Clinical and Laboratory Standards Institute (CLSI) criteria. Because the CLSI guidelines do not include interpretive criteria for some of the pathogens that we have tested, breakpoints for *Enterobacteriaceae*, mostly the *Escherichia coli* were adopted. The lists of these antibiotics for different lineages are provided in Supl. Table S4. Sensitivity or resistance to each antibiotic was determined by measuring the annular radius of inhibition of growth around each disc in accordance with the Clinical and Laboratory Standards Institutes (CLSI-2014). *E. coli* ATCC 25922 and *V. cholerae* O395 strains were used as a control in antimicrobial susceptibility tests. MIC for each drug was tested at a range of 1.5–160 μg/ml (Supl. Table S5D). Details of the test are mentioned in the Supl. Info. S1.

### Next generation DNA sequencing

The Genomic DNA from all the XDR enteric pathogens were prepared by using Cetyltrimethylammonium bromide (CTAB) method followed by RNase treatment. The quality and quantity of genomic DNA from 27 XDR pathogens were estimated using Biospectrophotometer (Eppendroff, Germany) and 0.8% agarose gel electrophoresis. Samples with OD_260_/OD_280_ ratio ≥1.8 and DNA concentration ~500ng/μl with no visible contaminating stable RNA or DNA degradation, were used for whole genome sequencing using 454 GS FLX+sequencing chemistry (Roche, USA). Details of the sequencing are provided in the Supl. Info. S1.

### Genome assembly, annotation and functional analysis

Filtered sequencing reads were used for genome assembly using *GS de novo genome assembler* (Brandford, CT, USA). First, seeds were generated from each read and assembly was done using minimum 40 nt overlap with more than 90% identity. Scaffolds were validated using MegaBLAST program. The Scaffolds for the genome were provided as inputs to the RAST server (version 7.3). The detailed annotations obtained for the genomes of six XDR isolates are provided in the Table S3. The protein ORFs obtained for the genomes were cross-compared with bacterial genomes isolated from various human body sites and draft sequenced as part of the Human Microbiome Project.

For the purpose of profiling the resistance genes in the genome, the ORFs were compared with the genes of the Antibiotic Resistance Genes Database(29), with thresholds of identity >70% and subject coverage >90%. All the predicted genes encoding enzymes that inactivate specific antibiotics either by hydrolysis or converting inactive derivatives by transferring specific functions were cloned into expression vectors and functional validation was done in homologous and heterologous genetic background (Supl. Table S5C).

### Genetic manipulations

The *hapR* positive *V. cholerae* O1 El Tor strain N16961 derivative SB8 was constructed by sequential allele replacement methods (Supl. Info. S1 **)**. Recombinant vector pAP5 was used to engineer the strain SB6 (Supl. Table S5B). Recombinant vector pAP6 was used to introduce the functional *hapR* gene in SB6 and create the strain SB8 (Supl. Table S5B). Genotype and phenotype of *hapR*+ derivative SB8 was confirmed by PCR and protease positive assays (Supl. Fig. S2).

All the antibiotic resistance genes with putative enzymatic functions were amplified from the genome of different XDR isolates and cloned under the control of P_BAD_ promoter in pBD62 or pBAD24 expression vectors (Supl. Table S5C). Specific primers were designed using the sequenced genomic information of respective XDR isolates (Supl. Table S5E). Restriction enzyme digestions and DNA sequencing confirmed recombinant vectors carrying different resistance genes.

### Conjugation and natural transformation

Rubidium chloride treated *E. coli* cells were used for transformation. Conjugation was done between diaminopimelic acid (DAP) auxotroph *E. coli* β2163 donors and wild type or mutant *V. cholerae* cells on 0.22-μm sterile filter paper. Detail methodology is provided in the Supl. Info. S1.

### Statistical analysis

Cross comparison of resistance trends across different groups was performed with Mann Whitney U tests using the ‘wilcox.test’ function implemented in the R programming package v3.0.0. For examining the association patterns of resistances to specific antibiotics in specific pathogenic species, the isolates were first grouped based on their species annotations. Subsequently association testing was performed using chi-square tests of independence. Subsequently association testing was performed using Fisher’s Exact Test (fisher.test function of R v3.0.0). Details are provided in the Supl. Info. S1.

### Nucleotide sequence accession number

16S rRNA gene sequences of all the six XDR isolates were deposited in the GenBank under the accession numbers KX302888.1, KX302887.1, KX302886.1, KX302885.1, KX302884.1 and KX302883.1. Sequences of all the annotated genes, translated proteins and relevant information of the genomes of these XDR isolates are provided in the Table S3. Complete genome sequences of all the XDR isolates were deposited in the Rapid Annotation using Subsystem Technology (RAST) database and will be available upon acceptance of the article.

### Ethical Clearance

Clinical samples (stool) were collected after obtaining informed consent from all the study subjects and approval from Institutional Ethical Committees, MVID Hospital, Delhi (No 1120/MS/MVIDH/2015) and NICED, Kolkata. Recombinant DNA works including DNA sequencing were carried out in accordance with the approved guidelines of Translational Health Science and Technology Institute Biosafety Committee. All methods were performed in accordance with the relevant guidelines and standard operating procedure (SOP) of the Centre for Human Microbial Ecology (CHME), Translational Health Science and Technology Institute.

## Electronic supplementary material

Supplementary information S1

Supplementary Table S1

Supplementary Table S2

Supplementary Table S3

Supplementary Table S4
