# Supplementary material for: Molecular Insights into Antimicrobial Resistance Traits of Multidrug Resistant Enteric Pathogens isolated from India
Source: Sci Rep. 2017 Oct 31;7:14468. doi: 10.1038/s41598-017-14791-1 (PMC5663842; doi:10.1038/s41598-017-14791-1)
Supplement: Supplementary file 1 — Supplementary information S1 [file 41598_2017_14791_MOESM1_ESM.pdf]

Title: Molecular Insights into Antimicrobial Resistance Traits of Multidrug Resistant Enteric Pathogens isolated from India

Pawan Kumar[1]*, Satyabrata Bag[1]*, Tarini Shankar Ghosh[1]*, Prasanta Dey[1]*, Mayanka Dayal[1], Bipasa Saha[1], Jyoti Verma[1], Archana Pant[1,2], Shruti Saxena[1], Anbumani Desigamani[1], Preety Rana[1], Dhirendra Kumar[3], Naresh C Sharma[3], Pranita Hanpude[4], Tushar K Maiti[4], Asish K Mukhopadhyay[5], Rupak K Bhadra[6], G Balakrish Nair[1,7], Thandavarayan Ramamurthy[1], Bhabatosh Das[1,3@]

Supplementary information S1

1. Bacterial species isolated and used for antibiotic susceptibility test in this study

Aeromonas spp, Escherichia coli, Klebsiella pneumonia, Providencia stuartii, Pseudomonas aeruginosa, Salmonella enterica Group (*AbonyII, Agona, Bareilly, Bovismorbificans, Denver, Emek, Enteritidis, Give, Hiduddify, Hindmarsh, Idikan, Infantis, Kentucky,* Kingston, Litchfield, Newport, Oakey II, Ohio, Stanley, Typhi, *Salmonella,* Virchow, Wangata, Weltevreden, Westhampton, Westminster) *Salmonella spp, Shigella boydii 12, Shigella dysenteriae 12, Shigella flexneri 1b, Shigella sonnei, Vibrio fluvialis* and *Vibrio parahaemolyticus.*

2. Preferred antibiotics for the treatment of relevant pathogens

K. pneumoniae: Ceftriaxone, Ciprofloxacin, Levofloxacin, Imipenem, Colistin

P. aeruginosa: Piperacillin, Colistin, Tigecycline, Ciprofloxacin, Fosfomycin

S. dysentery: Ciprofloxacin, Pivmecillinam, Ceftriaxone

S. Typhimurium*:* Amoxicillin, Ampicillin, co-Trimoxazole

Enterotoxigenic *E. coli*: Ampicillin, Cephalosporin, Tetracycline, Sulphonamides

3. Antibiotic susceptibility tests

For the disc diffusion method, exponentially growing bacterial cells were seeded on Muller-Hinton agar (MHA, Difco, USA) plate (23” x 23” cm) using sterile cotton swabs and commercially available discs (BD, USA) containing defined amounts of interested antibiotics were placed on it. Plates were incubated overnight at 37°C in static incubator.

In the broth dilution method, cells were grown to log phage in Luria- Bertani(LB) broth or Brain Heart Infusion (BHI) media. Antibiotics were dissolved in water or organic solvents as per guidance of manufacturer (Sigma, USA). The zone of inhibition and minimum inhibitory concentration (MIC) of antibiotics for different bacterial strains were selected based on the previous reports (M45-2015, M45-2016, M100S-2016 and M100-S24-2014). MIC was used as the lowest concentration to completely inhibit visible growth of selected isolates after 24 h incubation at 37°C.

4. Next generation DNA sequencing

Genomic DNA from each of the XDR pathogens was used for rapid library preparation by adopting GS FLX+ sequencing chemistry (Roche, USA). Briefly, about 1 µg of genomic DNA was nebulized by nitrogen gas at a pressure of 30 psi for

2 minute for obtaining DNA fragments of length 900-1500 base pairs (bps). The nebulized DNA was purified by PCR Purification kit (Qiagen, USA), end polished with T4 DNA Polymerase, Polynucleotide Kinase and Taq DNA Polymerase (Roche, USA). Reactions were performed at 25ºC for 20 minutes and 72ºC for 20 minutes. Sequencing adaptor oligonucleotides was ligated to the polished DNA fragments using DNA Ligase (Roche, USA). Ligation reaction was performed at 25ºC for 10 minutes. The genomic DNA libraries thus obtained were then purified by commercially available Ampure XP cleanup kit (Beckman Coulter, USA). The quality of DNA libraries was analyzed using DNA High Sensitivity Chip in 2100 Bioanalyzer (Agilent, USA). The total amounts of dsDNA in the libraries were quantified using Picogreen dye in QubitFluorometer (Invitrogen, USA). About 140 x 10^7 DNA library molecules per sample were clonally amplified by emulsion PCR in Mastercycler proS PCR systems (Eppendorf, Germany), purified using REMe integration (Roche, USA) on Biomek 3000 (Beckman Coulter, USA) and pyrosequenced in pico-titre plates in GS-FLX+ Genome Sequencers (Roche, USA). Sequence reads in FASTQ format were obtained from SFF files using sffinfo (GS-FLX) and a Linux script developed in-house. The sequence data generated per run were evaluated by FASTQC (http://www.bioinformatics.babraham.ac.uk/projects/fastqc/), using default parameters. In order to retain only the high quality reads in each metagenome, sequence reads having lengths less than 100 bps and at least one base with PHRAP quality scores less than 20, were removed from the metagenomic datasets.

5. Statistical analysis

For each association testing (resistance to a specific antibiotic in a specific pathogen or sampling center), 2X2 contingency tables were constructed using the four sets of values, namely the number of isolates having resistance to a given antibiotic in a group of interest (i.e. isolates from a specific species or sampled from a given center); number of isolates not having the corresponding resistance in that given group; number of isolates having resistance to the antibiotic but not belonging to the given group and; the number of isolates not having resistance to the antibiotic and not belonging to the concerned group. These values were then given as input to the 'chisq.test' functionality of R package v3.0.0 to obtain the statistical significance of the associations. Only those associations having a p-value of less than 0.05 were then reported.

Furthermore, the correlation patterns across resistances were obtained by first calculating the detection percentages of the resistances to various antibiotics in the isolates belonging to the different pathogenic species across the different years and then computing the Pearson correlations of these detection percentages across the different years (using core functionality of R package v3.0.0).

6. Engineering *hapR* positive whole genome sequenced *V. cholerae* cell

First, N16961Δ*hapR*::*ble* strain SB6 was engineered by using recombinant vector pAP5, a derivative of suicide plasmid pKAS32 carrying *sh ble* gene flanked by the upstream and downstream region of *hapR* allele. Then, Δ*hapR*::*ble* allele was replaced from the chromosome of SB6 using recombinant vector pAP6, derivative of pKAS32 carrying functional *hapR* allele from *V. cholerae* strains C6709. The gene replacement method was adopted as described previously (Das et al, 2009). Genotype and phenotype of *hapR* positive strains SB8 were confirmed by PCR amplification and HA protease activity measurement (Supl. Fig. S2) as reported previously (Syngkon et al. 2010).

References:

1. Das B, Pal RR, Bag S, Bhadra RK. Stringent response in *Vibrio cholerae*: genetic analysis of spoT gene function and identification of a novel (p)ppGpp synthetase gene. *Mol Microbiol* 2009; **72**(2): 380-98.
2. Syngkon A, Elluri S, Koley H, et al. Studies on a novel serine protease of a DeltahapADeltaprtV Vibrio cholerae O1 strain and its role in hemorrhagic response in the rabbit ileal loop model. *PloS one* 2010; **5**(9).

Legends to the supplementary information:

Supl. Information S1: Details of the materials and methods including isolation and cultivation of bacterial species, antibiotic susceptibility tests, genomic engineering, next generation DNA sequencing, genome assembly, gene annotations, brief description about the relevant bacterial strains and mobile genetic elements, plasmid curing experiment and statistical analysis used in this study are provided in the supplementary information S1.

Supl. Table S1: Name of the bacterial isolates, isolation ID, date of isolation and susceptibility to different antibiotics are mentioned in the supplementary Table S1.

Supl. Table S2: Resistance profile of 27 XDR isolates against 22 antibiotics representing nine distinct drug classes are mentioned in the supplementary Table S2. Whole genome sequenced XDR isolates are highlighted in boldface.

Supl. Table S3: Genomic information of six whole genome sequenced XDR pathogens.

Supl. Table S4: List of antibiotics (for different species) for which zone of inhibition and minimum inhibitory concentration interpretive standards are available or not available in CLSI.

Supl. Table S5: Details of the plasmids, antibiotic concentration, functional evaluation of resistance genes, primer sequences, β-lactamase enzyme classes are provided in the supplementary Table S5.

Supplementary Table S 5A: Several β-lactamase enzymes detected in the sequenced genome of XDR isolates

(i). β-lactamase enzymes present in the genome of XDR *Providencia stuartii* MV493

**Name**	**Size (aa)**	**Class**	**Closest member**
Beta-lactamase (EC 3.5.2.6)	380	C	*Providencia rettgeri* (Id, 92%; Sim 96%)
Beta-lactamase (EC 3.5.2.6)	381	C	*Salmonella enterica* (Id, 99%; Sim 100%)
Beta-lactamase (EC 3.5.2.6)	295	A	*Escherichia coli* (Id, 100%, Sim, 100%)
Beta-lactamase	270	B	*Acinetobacter baumannii* (Id, 100%, Sim, 100%)

(ii). β-lactamase enzymes present in the genome of XDR *Klebsiella pneumoniae* MV36808

**Name**	**Size (aa)**	**Class**	**Closest member**
β-lactamase (EC 3.5.2.6)	286	A	*Enterobacteriaceae* (Id, 100%; Sim, 100%)
β-lactamase	428	C	*Enterobacteriaceae* (Id, 99%; Sim, 99%)
β-lactamase	286	A	*Escherichia coli* (Id, 100%; Sim, 100%)
β-lactamase	270	B	*Acinetobacter baumannii* (Id, 100%; Sim, 100%)
β-lactamase	338	C	*Enterobacter hormaechei* (Id, 100%; Sim, 100%)
β-lactamase	291	A	*Pantoea agglomerans* (Id, 100%; Sim, 100%)
β-lactamase	295	A	*Escherichia coli* (Id, 100%; Sim, 100%)
β-lactamase	291	D	*Escherichia coli* (Id, 99%; Sim, 99%)

(iii). β-lactamase enzymes present in the genome of XDR *Pseudomonas aeruginosa* 36846

**Name**	**Size (aa)**	**Class**	**Closest member**
β-lactamase	610	C	*Pseudomonas sp.* (Id 98%, Si 98%)
β-lactamase	262	D	*Pseudomonas aeruginosa* (Id 100%, Si 100%)
β-lactamase	371	C	*Pseudomonas otitidis* (Id 99%, Si 99%)

β-lactamase	397	C	*Pseudomonas aeruginosa* (Id 99%, Si 100%)
β-lactamase	381	C	*Pseudomonas aeruginosa* (Id 99%, Si 99%)
β-lactamase	391	C	*Pseudomonas aeruginosa* (Id 99%, Si 100%)

(iv). β-lactamase enzymes present in the genome of XDR *Escherichia coli* MV292587

Name	Size (aa)	Class	Closest member
β-lactamase	377	C	*E. coli* (Id 100%, Si 100%)
β-lactamase	433	C	*E. coli* (Id 100%, Si 100%)
β-lactamase	286	A	*Klebsiella pneumoniae* (Id 100%, Si 100%)
β-lactamase	291	A	*Pantoea agglomerans* (Id 99%, Si 100%)
β-lactamase	291	D	*Serratia marcescens* (Id 99%, Si 99%)

(v). β-lactamase enzymes present in the genome of XDR *Shigella flexneri* MV07210

Name	Size (aa)	Class	Closest member
β-lactamase	376	C	*Shigella flexneri* (Id 99%, Si99%)
β-lactamase	291	D	*Serratia marcescens* (Id 99%, Si 99%)
β-lactamase	249	A	*E. coli* (Id 99%, Sim 99%)

(vi). β-lactamase enzymes present in the genome of XDR *Salmonella* Typhimurium MV32691

Name	Size (aa)	Class	Closest member
β-lactamase	293	B	*Salmonella enterica* (Id 100%, Si 100%)
β-lactamase	432	C	*Salmonella enterica* (Id 100%, Si 100%)

Supplementary Table S 5B: Relevant plasmids used in this study

Plasmids	Genotype/Phenotype	References

pBD62	pSW23T *sh ble ori*R6K, *mob*RP4, $attP^{ctx}$, Zeo^R	Das et al. 2014
pBAD24	pBR322 *ori; araC, bla;* Amp^R	Das et al. 2014
pKAS32	*Ori*R6K, *mob*RP4, *rpsL, bla*, conjugative vector; Amp^R	Das et al. 2009
pAP3	pUC18:: *hapR* region, Amp^R	This study
pAP4	pUC18:: Up-Dw Δ*hapR:sh ble*	This study
pAP5	pKAS32::Up-Dw Δ*hapR:sh ble*	This study
pAP6	pKAS32:: *hapR* region, Amp^R	This study

Supplementary Table S 5C: Evaluation of function of predicted ORFs. All the ORFs with putative antimicrobial resistance functions were cloned under the control of P_{BAD} promoter in pBD62 and/or pBAD24 vector and resistance functions were examined in the complemented host by broth dilution and/or disk diffusion assays.

*Amp- Ampicillin; Azt- Aztreonam; Car- Carbenicillin; Far- Faropenem; Fos-Fosfomycin; Gen- Gentamycin: Imp-Imipenem; Kan-Kanamycin; Ksg- Kasugamycin; Neo- Neomycin; Pen-Penicillin; PB -Polymyxin B; Spt-Spectinomycin; Str-streptomycin Tet-Tetracycline; Zeo- Zeocin
RE- Restriction endonuclease

**Name**	**Vector backbone**	**Insert**	**Host**	**Primers used**	**RE used**	**Function**
***K. pneumoneae* (MV36808)**						
pBIP27	pBD62	bla_{NDM1}	FCV14	681, 682	EcoRI, XbaI	Resistant: Amp25, Imp5.0<br>Sensitive: Azt1.6
***Providencia stuartii* (MV493)**						
pPD1	pBAD24	bla_{NDM}	FCV14	418, 419	EcoRI, PstI	Resistant: Imp 10<br>Sensitive: Azt1.6
pPD2	pFX497	bla_{NDM}	FCV14	443, 444	HindIII. SacI	Resistant: Imp 10<br>Sensitive: Azt1.6
pBIP10	pBIP11	Aph3	DH5α	458, 459	EcoRI, PstI	Resistant: Kan50, Gen1.5<br>Sensitive: Gen2.5/5 str50
pBIP14	pBIP11	*ksgA*	FCV14	452, 453	EcoRI, NsiI	Sensitive: Ksg50
pBIP15	pBIP11	*aph3'*	DH5α	454, 455	EcoRI, PstI	Sensitive: Kan50, Gen5, str50
pBIP16	pBIP11	*bl2be_shv2*	FCV14	456, 457	EcoRI, NsiI	Resistant: Amp100
pBIP17	pBIP11	bla_{NDM}	FCV14	Sub cloned from pPD 2	HindIII, SacI	Resistant: Amp100, Imp10<br>Sensitive: Azt1.6
pBIP18	pBIP11	*fosA*	FCV14	482, 483	HindIII, PstI	Sensitive: Fos4
pBIP19	pBIP11	*emrE*	FCV14	484, 485	EcoRI, PstI	Sensitive: Kan50, Gen5, str50
pBIP20	pBIP11	metallo-blb gene	FCV14	486, 487	EcoRI, PstI	Sensitive: Imp1
pBIP21	pBIP11	*tet34*	FCV14	490, 491	EcoRI, PstI	Sensitive: Tet 1.5

Supplementary Table S 5D: List of antibiotics, abbreviation and minimal inhibitory concentration (MIC) used in this study

Antibiotic	Chemical class	Antibiotic abbreviation	MIC	
			Solution (μg/ml)	Disc
Ampicillin	β-lactam	Amp	10	10
Penicillin	β-lactam	Pen	-	10
Carbenicillin	β-lactam	Car	-	100
Imipenem	β-lactam	Imp	10	10
Faropenem	β-lactam	Far	4/10/130	-
Aztreonam	β-lactam	Azt	1.6	-
Polymixin B	polypeptide	PB	50	300
Kanamycin	Aminoglycoside	Kan	30/40	30
Spectinomycin	Aminoglycoside	Spt	40	15
Streptomycin	Aminoglycoside	Str	100	10
Neomycin	Aminoglycoside	Neo	40	40
Gentamicin	Aminoglycoside	Gen	10	-
Chloramphenicol	Chloramphenicol	Chl	3/30	30
Tetracycline	Tetracycline	Tet	1.5/5	5
Doxycycline	Tetracycline	Dox	-	30
Erythromycin	Macrolide	Ery	100	100
Rifampin	Ansamycins	Rif	2/5	5
Ciprofloxacin	Quinolones	Cip	4/30	5
Nalidixic acid	Quinolones	Nal	4/30	30
Zeocin	Glycopeptide	Zeo	25	-
SXT	Sulfonamide-Pyrimidine	SXT	-	Sulfamethoxazole 23.75 Trimethoprim 1.25
Trimethoprim	Pyrimidine	Tmp	30	-
Sulfamethoxazole	Sulfonamide	Sss	160	-

Supplementary Table S 5E: Primers used in this study.

Primer Name	Primer sequence	Target gene
418	CCGAATTCACCATGGAATTGCCCAATATTATG	bla_{NDM} *P. stuartii*
419	GGCTGCAGTCAGCGCAGCTTGTCGGCCATG	bla_{NDM} *P. stuartii*
428	GCGAGCTCTGGTGGTTCATAGCCATGAG	*hapR* gene region of C6709
429	CCTCTAGAATCATCGCCTACTGTGTC	*hapR* gene region of C6709
443	GGAAGCTTTGACCTATGACCGTGGACGTGAG	bla_{NDM} gene of *P. stuartii*
444	GGGAGCTCTCAGCGCAGCTTGTCGGCCATG	bla_{NDM} gene of *P. stuartii*
448	CCGAATTCGTCACATTCAATTCAATTCC	*bla* gene of *P. stuartii*
449	GGCTGCAGGTATTACTGGTTCGTTATCTG	*bla* gene of *P. stuartii*
450	GGGGATCCGATTACGGTTGTATGCTACG	Polymyxin resistance gene of *P. stuartii*
451	CCCTGCAGCCTTGGTAGGTATCCACATC	Polymyxin resistance gene of *P. stuartii*
452	CCGAATTCCCACGACACATCTGCCACTC	kasugamycin resistance gene of *P. stuartii*

453	CCATGCATCGCGCAATATCGGGTTGAG	kasugamycin resistance gene of *P. stuartii*
454	CCGAATTCGCTTATAGCAGTGTCACAG	*aph* gene of *P. stuartii*
455	CCCTGCAGGGTGGTTTATGTCGCACTTC	*aph* gene of *P. stuartii*
456	CCGAATTCGGAAATTGCTCATCAGCTCAG	*bla* gene of *P. stuartii*
457	GGATGCAGGAGTAAACTTGGTCTGACAG	*bla* gene of *P. stuartii*
458	CCGAATTCCCTCTGATGTTACATTGCAC	*aph* gene of *P. stuartii*
459	CCCTGCAGGCTCTGCCAGTGTTACAACC	*aph* gene of *P. stuartii*
482	GGCTGCAGGGAGGTAGGTGGTTACGATACTG	*fosA* gene of *P. stuartii*
483	CCAAGCTTTATACAACTCTGCGTAAAGC	*fosA* gene of *P. stuartii*
484	GGGAATTCCAGATGCCACCGACTTGGCAATG	*emre* gene of *P. stuartii*
485	CCCTGCAGCTGCCTGAATATTCTGCACC	*emre* gene of *P. stuartii*
486	GGCTGCAGCGTTGCGAAGAAATGACAAG	bla_{NDM1} gene of *P. stuartii*
487	CCGAATTCCTCGTGAATTGCAGTCGTCC	bla_{NDM1} gene of *P. stuartii*
490	CCGAATTCGACCGAGCAACTCAAATTAC	*tet34* gene of *P. stuartii*
491	GGCTGCAGGTCTATCGGAGTTGAATTTGG	*tet34* gene of *P. stuartii*
492	GGGAATTCGCAAGGTAGCCAACTAAAGG	*rosa* gene of *P. stuartii*
493	CCCTGCAGGAGATCGTCATCAAATAATTAACC	*rosa* gene of *P. stuartii*
681	CCGAATTCATGAAATTATCTGCCCTTGC	bla_{NDM} gene of *K. pneumoneae*
682	CCTCTAGATCACCACTTCATCTCACC	bla_{NDM} gene of *K. pneumoneae*

Supplementary Figures

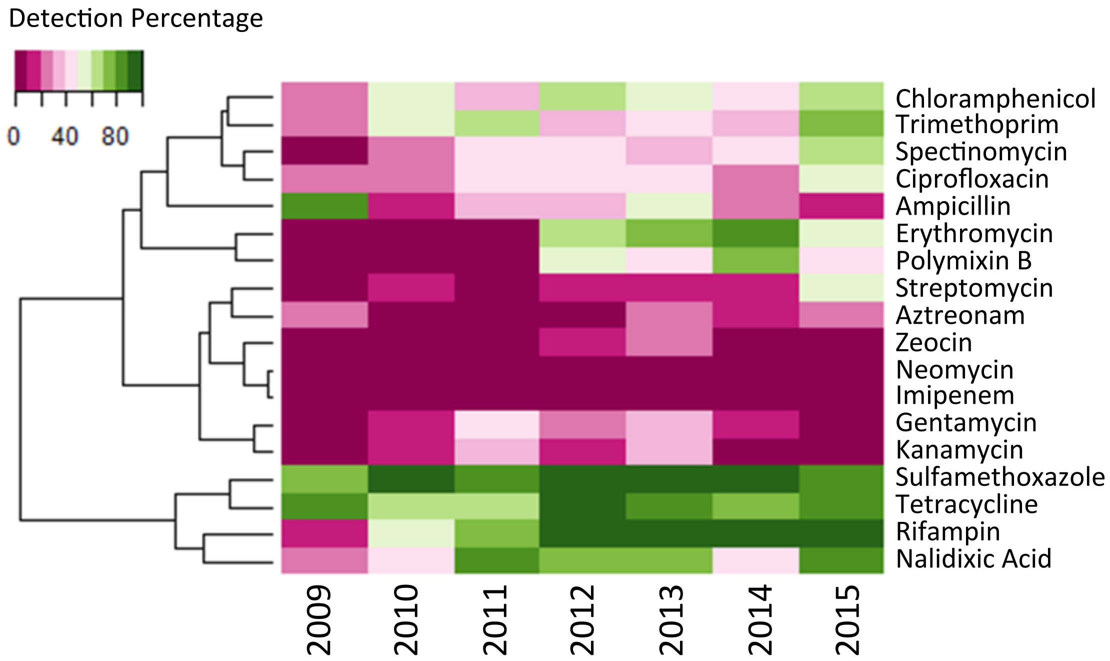

Supl. Figure S1: Detection percentage of resistance to various antibiotics across the years 2009-2015.

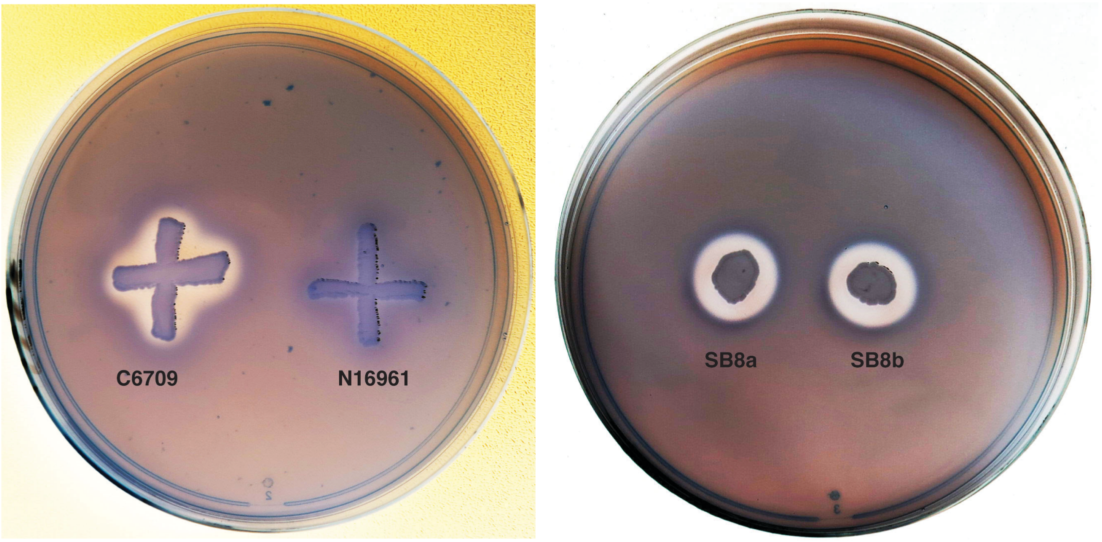

Supl. Figure S2: Protease assay of *hapR* positive *V. cholerae* strains SB8 derived from whole genome sequenced strain N16961. Zone of clearance of skim milk protein in the minimal medium indicate expression and secretion of extracellular protease modulated by transcriptional factor HapR.

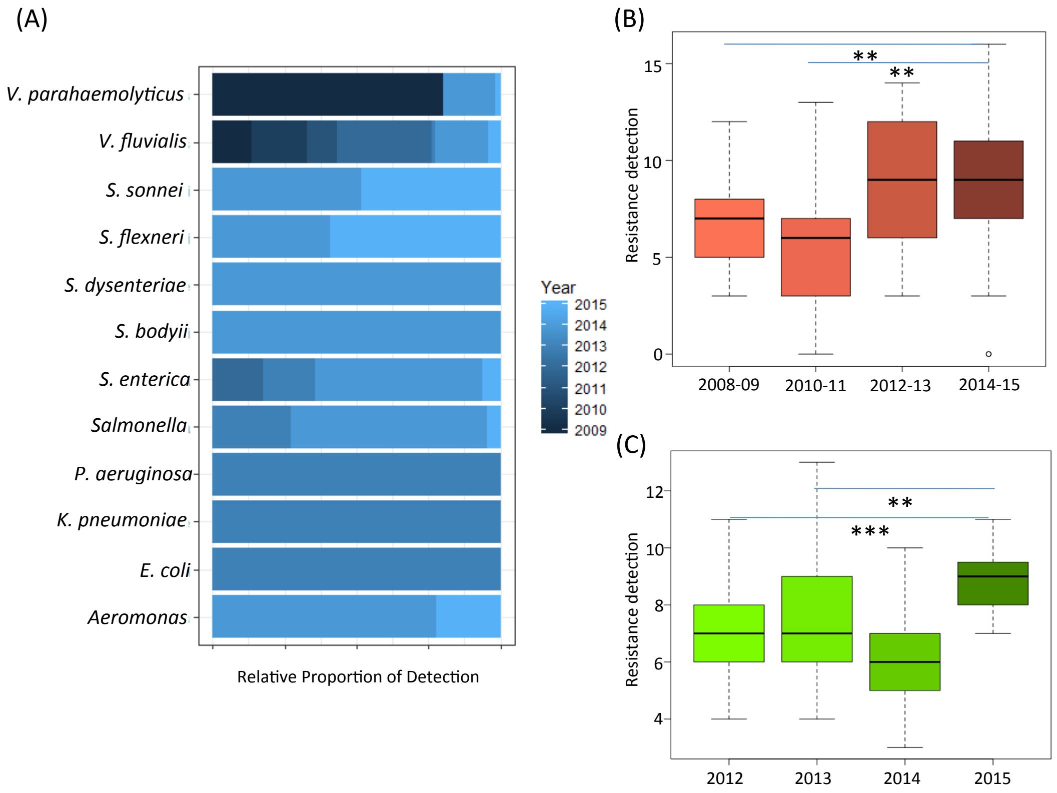

Supl. Figure S3: (A): Detection pattern of various pathogens across the years from 2009-2015 (B) Resistance detection (i.e. the number of antibiotics against which resistance was detected) of the isolates of *V. fluvialis* across the years. (C) The number of antibiotics against which resistance was detected for the isolates of *S. enterica* obtained from 2012-15. Significant differences between the current and earlier years are highlighted with *s. ** indicates P-value < 0.01 and *** indicates P-value < 0.001.
